# Clinicopathological findings and outcome of lupus nephritis in Tunisian children: a review of 43 patients

**DOI:** 10.11604/pamj.2017.27.153.10915

**Published:** 2017-06-30

**Authors:** Hela Jebali, Meriam Hajji, Lamia Rais, Fethi Ben Hamida, Soumaya Beji, Mohammed Karim Zouaghi

**Affiliations:** 1 Nephrology Departement, La Rabta Hospital, Tunis, Tunisia; 2 Laboratory of Kidney pathology LR00SP01, Charles Nicolle Hospital, Tunis, Tunisia

**Keywords:** systemic lupus erythematosus, nephropathy, children, renal biopsy

## Abstract

We report clinical and renal histological data, treatment modalities and outcome of 43 Tunisian children with biopsy-proven lupus nephritis seen over a 23-year period. There were 39 girls and 4 boys with a mean age of 12.5 years at diagnosis of lupus nephritis and followed for a mean period of 77 months. Renal symptoms included urinary abnormalities in all patients, hypertension in 40% of cases, nephrotic syndrome in 60% of cases and renal failure in 25% of cases. Class IV and class III nephritis were observed in 48.8 % and 30.2 % respectively. Corticosteroids were used in all cases, associated to immunosuppressive therapy in 23%. Overall survival was 86% at 5 years and 74% at 10 and 15 years. Renal survival was 83% at 5 and 10 years and 63% at 15 years. Initial renal failure and tubulointerstitial fibrosis were significantly increased risk for the development of end-stage renal disease in our study group. Renal histological findings provide the basis for treatment recommendations. Timely performed renal biopsy is greatly needed to accurately determine the prognosis and to guide treatment in children lupus nephritis.

## Introduction

Systemic lupus erythematosus (SLE) presents throughout the age spectrum and one of its most severe manifestations, lupus nephritis (LN), may have devastating consequences at any age [1]. Renal disease occurs in 20 % to 75% of all SLE patients, mostly within the first 2 years after diagnosis [1, 2]. SLE presenting in childhood (cSLE) accounts for 15 to 20% of all cases. It occurs in 15-20% before the age of 16 [1, 3]. There are until now no studies about LN in Tunisian children. Therefore, we conducted a retrospective study describing epidemiological, clinical, biological and pathological features of LN and their correlations as well as prognostic factors.

## Methods

The present study reports a cohort of 43 SLE patients with disease onset before the age of 16 year-old from January 1983 to December 2006. All patients fulfilled at least four of the American College of Rheumatology criteria for the classification of SLE [4]. We retrospectively reviewed the medical records of all children and analyzed the clinical and laboratory features at the time of renal biopsy which included age at onset of SLE and LN, gender, follow up duration, extra renal manifestations, Clinico-pathological data, treatment modalities and outcome. Laboratory data collected included complete blood count (CBC), urinalysis, 24 hours urine protein, serum creatinine and immunological investigations. Age at onset of disease was defined as the time of LN diagnosis. All patients satisfied diagnosis criteria for lupus nephritis which were increasing serum creatinine or confirmed proteinuria ≥1g in 24-h urin or proteinuria ≥0,5g in 24-h urin plus hematuria or proteinuria ≥0,5g in 24-h urin plus cellular casts.

Hypertension was defined as blood pressure greater than the 95^th^ percentile for age and sex [5]. Estimated glomerular filtration rate (eGFR) was estimated by the formula of Schwartz et al. [6] for eGFR. Renal failure was defined as eGFR less than 60 ml/min per 1.73m^2^. Nephrotic syndrome was defined as proteinuria greater than 50 mg/kg in 24-h urin and serum albumin less than 3 g/dl.

All patients underwent renal biopsy. Renal lesions were classified according to the World Health Organization classification (WHO) [7]: class II: pure mesangiale proliferative LN; class III: focal segmental proliferative glomerulonephritis LN; class IV: diffuse glomerulonephritis LN and class V: diffuse membranous glomerulonephritis LN. Mixed class IV + class V and class III+ class V was grouped as class IV and class III respectively. A second or even a third renal biopsy was in some cases indicated during the course of the disease. Specific histological features were assessed in each biopsy to give insight into activity and chronicity of lesions.

The duration of follow-up was calculated from the time of LN diagnosis until the patient’s last clinic visit before December 2006. Renal outcome was defined as: (1) complete remission: resolution of proteinuria and haematuria, serum creatinine and eGFR within normal limits, (2) partial remission: resolution of nephrotic syndrome, improvement in proteinuria less than 1 g /24 hours and > 50% improvement in serum creatinine level (or eGFR), (3) end stage renal disease (ESRD): eGFR less than 15 ml/min/m² or the need to start dialysis and (4) Relapse: presence of any of the following criteria in at least two determinations: (a) increase in proteinuria by more than 2 g/24 hours, (b) active urine sediment, or (c) increase in creatinine by> 30%.

**Statistical analysis:** Statistical analysis were based on Pearson‘s chi-square test and logistic regression analysis using SPSS soft-ware. A P value of less than 0,05 was considered statistically significant. Patient survival curves were plotted using the Kaplan and Meier survival curves and the log rank test was used for significance.

## Results

The study population consisted in 43 children with LN. There were 39 females and four males (The female to-male ratio: 9.75/1). The mean age at onset of lupus was 11.95 years (range: 6-16 years). The mean age at diagnosis of LN was 12.58 years (range: 3-19 years). The average time between renal biopsy and clinical diagnosis of LN was 7.35 months (0-60 months). Extra renal manifestations of SLE are summarized in (Table 1). Pericarditis, musculoskeletal and mucocutaneous involvement were the most common extra renal presentation, while neurological disorder was the least frequent manifestation.

**Table 1:** Extra-renal manifestations

Manifestations	No	(%)
**General**		
Fever	27	62.8
Fatigue	18	40
Anorexia	17	39.5
**Mucocutaneous**	**33**	**76.6**
Photosensitivity	16	37.2
Malar rash	28	65
Discoid rash	6	14
Oral ulceration	5	11.6
**Musculoskeletal**	**37**	**86**
Arthralgia	37	86
Arthritis	15	34.9
**Vascular**	**12**	**27.9**
Purpura	10	23.3
Livedo	2	4.7
**Serositis**	**28**	**65**
Pericarditis	19	67.8
Pleurisy	9	20.9
**Neurological disorder**	**6**	**13.9**

Hemolytic anemia, thrombocytopenia, leucopenia and lymphopenia were reported in 48%, 11.6%, 16.3% and 20.9% respectively. Regarding immunological characteristics of patients, a positive ANA test was found in 97.7% and a positive anti-dsDNA was found in 93%. On the other hand, low C3 and C4 were observed in 82 and 83 % respectively. Anti phospholipids antibodies were positive in 45%, but only three patients met the diagnostic criteria for antiphospholipid syndrome (Table 2).

**Table 2:** Laboratory findings

Parameters	No. ^+^/ N^++^	(%)
**Hematologic disorder**		
Anemia	25	58,1
hemolytic anemia	12	48
Leucopenia	7	16,3
Lymphopenia	9	20,9
Thrombocytopenia	5	11,6
Renal involvement	43	100
Proteinuria	43	100
Hematuria	39	90,6
Nephrotic syndrome	26	60,4
renal insufficiency	11	25,5
**Immunological findings**		
ANA	***43 /42***	97,7
Anti-DNA	43/40	93
Anti-Sm	17/12	70
Low C 3	39/32	82
Low C4	***37/31***	83
Lupus anticoagulant	8/4	50
Anticardiolipin	22/10	45

^+^Number of patients positive to the test ^++^Number of patients that were tested

Concerning renal manifestations, hematuria was present in 39 patients (90.6%), proteinuria in all patients, and 26 of them (60,4%) presented nephrotic syndrome. Hypertension was found in 17 patients (40%) before corticoids treatment. Mean creatinine was 93 µmol/l (39-434 µmol/l). 32 patients (74, 4 %) have normal renal function. Renal impairment was documented in 11 patients (25.5%). Mean creatinine in this group was 179 µmol/l (125-434 µmol/l) and mean eGFR was 43 ml/min per 1.73 m2. Three patients required dialysis at LN diagnosis. The most frequent histopathological finding was class IV LN ( 21;48.8%), followed by class III (13;30.2%), class V (5;11.6%) and class II (4;9.3%) LN. Class III was associated with class V in 5 patients (11.6 %) and class IV was associated with class V in 8 patients (18.6%). Proliferative lesions were found in 34 patients (79%). There was no significant difference in LN presentation between histopathological groups. In the other hand, there was no significant difference in chronicity and activity scores between proliferative classes. Table 3 summarize renal manifestations according LN classes. In addition, comparing immunological features according to LN classification showed that class IV LN was notably associated with Low C3 and anti-dsDNA antibodies (90.4%), while patients with class V LN had the lowest rate of immunological abnormalities (low C3 in 40%, anti-dsDNA in 80%).

**Table 3:** Renal manifestations according to histologic classes

Histology classes	II (4)	III (13)	IV (21)	V (5)	P
Hypertension	0	5	10	2	NS
Renal impairment	0	4	8	0	NS
Nephrotic syndrome	0	7	14	5	NS
Mean creatinine (ymol/l)	50	81	108	71	NS
eGFR(ml/min)	120	109	90	104	NS
Anti-DNA	4	13	19	4	NS
Low C3	3	10	19	2	NS

NS: not significant

**Figure 1:**
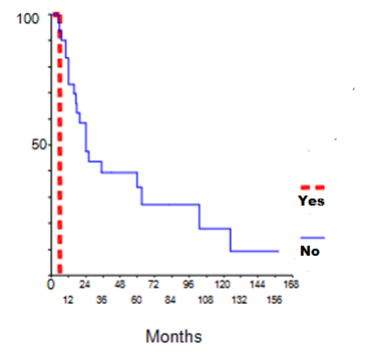
Rate of renal relapse by initial impaired renal function

**Figure 2:**
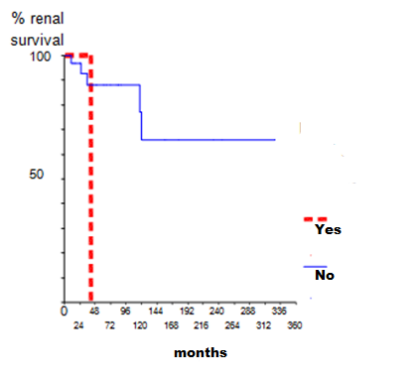
Overall renal survival by initial impaired renal function

**Figure 3:**
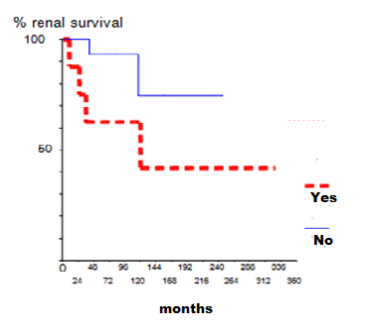
Overall renal survival by tubulointersitial fibrosis

Corticosteroids (Cs) were the commonest initial medications used in all patients at presentation. Ten patients were treated with intravenous pulse methylprednisolone. For patients with class III, IV and V, ten of them (23%) received immunosuppressive therapy in addition to steroids (Cyclophosphamide (CYC) in 8 patients and Azathioprine (AZA) in two cases). Maintained therapy consisted in low dose of Cs in all patients. Immunosuppressive therapy was associated with Cs in ten patients (CYC in 4 patients, AZA in 5 patients and mycophenolate mofetil in one case). Sixteen (37.2%) patients received Hydroxychloroquine. In our study, 35 out of 43 patients were followed up. Mean period of follow-up was 77 months ranging from 2 to 327 months. At the last follow-up, 16 (45.7%) patients were in complete remission, four (11.4%) in partial remission, while 15 patients (42.8%) had adverse outcome with chronic renal failure in 9 cases and ESRD in six patients. Nine patients (60%) with renal failure at the last follow-up had renal impairment at presentation. Five patients with ESRD at last follow-up, had proliferative lesions while one patient had class V. Twenty three patients had renal relapse (65.7%). We noted that non-adherence to treatment was the major cause for relapse (52.1%). Using univariate regression analysis, renal failure at presentation was the only predictor factor for the occurrence of relapse (p=0.03) (Figure 1). Second renal biopsy was performed in 13 patients. The indications for iterative biopsy were renal relapse in 11 patients and resistance to treatment in 2 patients. We showed the same class as that of the initial biopsy in 8 patients, while a conversion from class III to class IV was found in 4 patients and class IV to class III was observed in one patient. After therapy of renal relapse, seven patients were in complete remission, while four patients developed ESRD. Complications occurred in 27 patients (62%). Infection was the leading complication during treatment of LN (54%). De novo diabetes was occurred in five patients, ovarian failure and avascular necrosis were observed in three patients. Six patients died (17.1%), from septic shock in three cases, hyperkalemia in one case, lupic myocarditis and pulmonary embolism in the two other patients. The estimated global survival was 86% at five and ten years and 74 % at 15 years, while renal survival was 83% at 5 and 10 years and 63% at 15 years. We studied the factors influencing the survival of patients. We found that the presence of neuropsychiatric lupus, hypertension, infectious complications, proliferative lesions or ESRD were more frequent in the deceased group but without significant correlations while the probability of developing ESRD was statistically higher in patient with renal failure at presentation or tubulointerstitial fibrosis in renal biopsy (Figure 2 and Figure 3).

## Discussion

Our study illustrated clinicopathological findings, treatment and outcome of LN in Tunisian children suggesting that LN exhibits a severe clinical, biological and histological pattern when compared to other ethnic populations. The most important finding was the high rate of ESRD and mortality in children with LN. The other notable findings are the observation of high incidence of proliferative lesion (class III or IV LN) on renal biopsy. The present study has some limitations. It is a retrospective analysis. Because of the long follow-up period, in which patients were collected, treatment varied widely between patients. Despite its limitations, this current study represents, to our knowledge, the only pediatric LN cohort from Tunisia.

About 15 to 20% of SLE starts in childhood. However, the exact prevalence of cSLE among the SLE population remains unknown [2, 8]. In Tunisia, S.Yaalaoui [9] has reported 16 patients with cSLE during a period of 9 years. In Egypt, several studies [10, 11] have reported a relatively high prevalence of lupus in Egyptian children compared to Tunisian children. The disease was diagnosed before the age of 10 years in 17 % of patients. The majority of cSLE children were aged above 10 years [12, 13]. Children with SLE are more susceptible than adults to nephritis [10, 12]. In a study of S.Yaalaoui, renal involvement was found in 75 % of patients [9]. In Arab countries, pediatric lupus nephritis was noted in 29 to 80% of cases [13, 14]. In western countries, renal involvement was reported also in 30 to 80 % [15, 16].

In our study, female to-male ratio was 9.75/1 while in the other reports, there was a lower predilection for female gender in childhood lupus [17, 18]. Concerning renal manifestations, hematuria and proteinuria are the most commonly identified abnormalities, being reported in 67 to 100% of affected children in different series [8, 16]. In our group, proteinuria was observed in 97.7%, and nephrotic syndrome was found in 62.7 % of patients and this is slightly higher than in other studies [17]. Hypertension varies significantly across different studies, from 30 to 50 % [18, 19]. In our study, hypertension was found in 39 % of patients. The frequency of renal failure ranged from 20 to 70% [20]. In our study, renal failure at presentation was noted in 25.5% and this is significantly less common than in the study reported by S.Yaalaoui et al. (66.6%) [9]. When compared to Tunisian adult series of SLE [21], renal manifestations appear to be similar to our group. This result was also compatible with the findings reported by Mak et al [22].

In our study, The most frequent histopathological finding was class IV LN (48.8%), approaching the results from several reports, ranging from 37% to 46% [23, 24]. In our study, proliferative lesions were observed in 79% of cases and this result was similar to an italian study [25]. Class V LN has been reported to have a lower incidence in children [26]. In our group, pure class V LN was found only in 11.6% of cases. In our group, patients with class IV LN have more hypertension and renal failure at presentation, while nephrotic syndrome was more common in class V LN.

The treatment recommendations for childhood lupus nephritis have been extrapolated from adult studies or derived from past reports of case series. Thereby, new regimens using steroids in addition to lower doses and shorter treatment durations of intravenous CYC or MMF can been used in children with low toxicity without sacrificing efficacy of therapy [27]. In the other hand, rituximab can improve disease activity but controlled studies are required [28]. All of our patients received a higher initial steroids or equal to 1mg / kg / day (97.4%). Immunosuppressive therapy was administered in 23 % of cases at induction therapy.

The outcome for pediatric lupus nephritis has improved over the last 30 years with the addition of corticosteroids to cytotoxic agents [8, 17]. In our study group, complete remission and partial remission were observed in 45.7 % and 11.6% respectively after a mean period of six mouth of induction treatment. Several studies have reported a better rate of complete and partial remission, ranged from 30 to 80 % [20–28]. A possible explanation for our result was the delayed renal biopsy after clinical presentation, delayed administration of appropriate treatment and the use of steroids alone in the majority of cases. Relapse rate was 65.7% in our cohort. This funding is higher than the result of the most studies [27–30]. Renal flares were caused by non adherence to treatment in 51,6 %,while infectious complications were noted in 31,5 %. Our result and the report by Rivera F et al. [31] emphasize that non adherence to treatment in LN is a challenge for clinician, particularly in young patients. It has been reported that younger age, high activity index or chronicity index and delayed of initiation treatment were predictive of renal flares [15, 22]. In our study, only increased creatinine at presentation was associated with renal relapse and estimated renal survival was lower than the majority of pediatric series [13, 17, 20]. There was no difference in renal survival between different histological classes. However, tubulointerstitial fibrosis was associated with a poor renal outcome. The mortality rate in our study group was 17.1%. The mortality rate in other studies has varied from 5% to 30% [27–28].

## Conclusion

In conclusion, lupus nephritis is rare in children. The clinical-biological and histological characteristics of renal disease in our patients confirm its severity. The study of therapeutic modalities showed the absence of a clear and common approach between the various teams. Treatment should be tailored to the histological class. Adequate care must include regular monitoring, education of the child and his entourage about the disease and the importance of treatment.

### What is known about this topic

- Childhood-onset systemic lupus erythematosus (cSLE) is a severe multisystem autoimmune disease;
- Renal involvement occurs in the majority of cSLE patients and is often fatal;
- The outcome of lupus nephritis is primarily dependent on histological classification at presentation and aggressive immunosuppression therapy is recommended to improve mortality and morbidity of such patients.

### What this study adds

- The first cohort of lupus nephritis in children in Tunisia;
- LN exhibits a severe clinical, biological and histological pattern in our population when compared to other ethnic populations;
- The high incidence of proliferative lesion (class III or IV LN) on renal biopsy.

## Competing interests

The authors declare no competing insterests.
